# Topographical Organization of M-Current on Dorsal and Median Raphe Serotonergic Neurons

**DOI:** 10.3389/fncel.2021.614947

**Published:** 2021-02-25

**Authors:** Tsogbadrakh Bayasgalan, Andrea Csemer, Adrienn Kovacs, Krisztina Pocsai, Balazs Pal

**Affiliations:** Department of Physiology, Faculty of Medicine, University of Debrecen, Debrecen, Hungary

**Keywords:** M-current, dorsal raphe (DR), median raphe (MR), serotonergic neuron, firing pattern, spike frequency adaptation

## Abstract

Dorsal and median raphe nuclei (DR and MR, respectively) are members of the reticular activating system and play important role in the regulation of the sleep-wakefulness cycle, movement, and affective states. M-current is a voltage-gated potassium current under the control of neuromodulatory mechanisms setting neuronal excitability. Our goal was to determine the proportion of DR and MR serotonergic neurons possessing M-current and whether they are organized topographically. Electrophysiological parameters of raphe serotonergic neurons influenced by this current were also investigated. We performed slice electrophysiology on genetically identified serotonergic neurons. Neurons with M-current are located rostrally in the DR and dorsally in the MR. M-current determines firing rate, afterhyperpolarization amplitude, and adaptation index (AI) of these neurons, but does not affect input resistance, action potential width, and high threshold oscillations.These findings indicate that M-current has a strong impact on firing properties of certain serotonergic neuronal subpopulations and it might serve as an effective contributor to cholinergic and local serotonergic neuromodulatory actions.

## Introduction

DR and MR are members of the reticular activating system and play important role in the regulation of sleep-wakefulness cycles, movement, and affective states. The activity of DR is increased during wakefulness, meanwhile, it is less active in paradoxical or REM-sleep (Monti, 2011).

M-current is present in several brain areas including the brainstem (Kharkovets et al., 2000; Koyama and Appel, 2006; Hansen et al., 2008). It is a voltage-gated potassium channel modulated by metabotropic receptors; and inhibited by the activation of muscarinic acetylcholine receptor and other G-protein coupled receptors like 5HT_2C_ receptor (Marrion, 1997; Roepke et al., 2012).

It was shown that DR and MR serotonergic neurons have several subgroups based on expressed markers, morphological characteristics, and *in vitro* and *in vivo* electrophysiological properties (Hornung, 2003; Andrade and Haj-Dahmane, 2013). Approximately 60% of DR serotonergic neurons express KCNQ4, the main ion channel subunit for M-current (Hansen et al., 2008; Zhao et al., 2017). However, neither the proportion of MR serotonergic neurons possessing M-current nor the topographical distribution of these neurons in both nuclei, as well as their contribution to distinct electrophysiological parameters of the serotonergic neurons has been determined yet.

It is known from previous publications that the M-current contributes to the action potential after afterhyperpolarization and spike frequency adaptation regulates the resting membrane potential and controls presynaptic functions (Koyama and Appel, 2006; Tzingounis et al., 2010; Huang and Trussell, 2011; Nigro et al., 2014). It also modulates neuronal membrane potential oscillations (Bordás et al., 2015).

Here we provide evidence that serotonergic neurons possessing M-current can be found both rather in the rostral DR and the dorsal MR. M-current of raphe serotonergic neurons determines firing frequency, spike frequency adaptation, and AHP amplitude, but does not affect input resistance, action potential width, delay of the first action potential, and oscillatory activity.

## Materials and Methods

Slice electrophysiological experiments were conducted in artificial cerebrospinal fluid (aCSF) with the following composition (in mM): NaCl, 120; NaHCO_3_, 26; glucose, 10; KCl, 2.5; myo-inositol, 3; sodium-pyruvate, 2; NaH_2_PO_4_, 1.25; ascorbic acid, 0.5; CaCl_2_, 2; MgCl_2_, 1; pH 7.3. Low Na^+^ aCSF was used for preparation, while 95 mM NaCl was replaced by glycerol (60 mm) and sucrose (130 mm). All chemicals were purchased from Sigma–Aldrich (St. Louis, MO, USA), unless otherwise stated.

Animal experiments were performed according to the appropriate national, international (EU Directive 2010/63/EU for animal experiments) and institutional guidelines and laws on the care of research animals (Committee of Animal Research of the University of Debrecen; 5/2015/DEMÁB; 8/2015/DEMÁB; 19/2019/DEMÁB). 12–22 days old mice expressing tdTomato fluorescent protein in a Serotonin transporter- (Sert) dependent way were used from both sexes (*n* = 26). Obtaining these mice, homozygous floxed-stop- tdTomato [B6;129S6-Gt(ROSA)26Sor^tm9(CAG-tdTomato)Hze/^J; Jax mice accession number: 007905] and Sert-cre [B6.129(Cg)-Slc6a4tm1(cre)Xz/J; Jax number: 014554] strains (Jackson Laboratories, Bar Harbor, ME, USA) were crossed. Midbrain coronal slices (200 μm thickness) were prepared in ice-cold (0 to −2°C) low Na^+^ aCSF with a Microm HM 650V vibratome (Microm International GmbH, Walldorf, Germany). Slices were incubated in normal aCSF for 1 h at 37°C before the experiment.

Patch pipettes with 6–8 MΩ resistance were filled with the following internal solution (in mM): K-gluconate, 120; NaCl, 5; 4-(2-hydroxyethyl)-1-piperazineethanesulfonic acid (HEPES), 10; Na_2_-phosphocreatine, 10; EGTA, 2; Mg-ATP, 5; Na_3_-GTP, 0.3; CaCl_2_, 0.1; biocytin, 8; pH 7.3. Whole-cell patch-clamp experiments were performed at room temperature (24–26°C) with an Axopatch 200A amplifier (Molecular Devices, Union City, CA, USA). Clampex 10.0 software was used for data acquisition, whereas data analysis was performed by Clampfit 10.0 software (Molecular Devices, Union City, CA, USA). Only stable recordings with minimal leak currents were considered, with series resistance below 30 MΩ and less than 10% change of the series resistance was tolerated. To eliminate action potential firing, 1 μM tetrodotoxin was used (TTX; Alomone Laboratories, Jerusalem, Israel).

**Figure 1:**
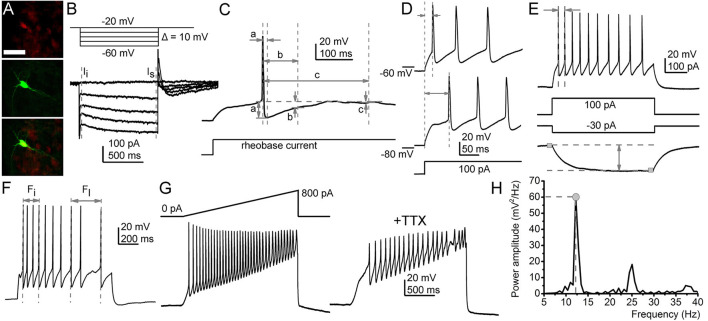
Functional parameters recorded in this study. **(A)** Identification of the serotonergic neurons. Sert-driven tdTomato expression (red, upper panel), biocytin labeling (green, middle panel), merged image (bottom panel). Scale bar: 50 μm. **(B)** A representative M-current trace (below) in the voltage protocol eliciting it (above). Gray dashed lines: instantaneous (I_i_) and steady-state (I_s_) currents. The M-current was defined as the difference between them. **(C)** Afterhyperpolarization amplitudes (AHP) recorded with square rheobase current (20–50 pA; a. maximal AHP; b. medium AHP at 100 ms from the action potential peak; c. slow AHP at 300 ms). **(D)** Determination of the first action potential delay at 100 pA depolarizing square current injection (from membrane potentials set to −60 and −80 mV). **(E)** Maximal and average firing rate and the input resistance. (Top) Voltage trace elicited by 100 pA depolarizing square current injection. Arrows: period used for calculation of maximal firing rate. (Bottom) Voltage trace elicited with 30 pA hyperpolarizing square current injection. Gray squares: 20 ms-long segments used for calculation of the voltage difference elicited by the hyperpolarizing current injection. **(F)** Calculation of the adaptation index (AI). Arrows initial (F_i_) and last action potential (F_l_) frequencies used for calculation with the following equation: Adaptation index (AI) was calculated with the following equation: AI = 1 − (F_l_/F_i_). **(G,H)** Methods for assessing oscillatory activity. **(G)** Depolarizing ramp current injection (above left) and the voltage trace elicited by it (below left). The voltage trace with TTX (right). **(H)** The power spectrum of the TTX-resistant oscillatory activity. Gray circle, dashed lines: power maximum, frequency belonging to power maximum.

Details of voltage and current clamp protocols are shown in Figure 1 and based on our previous publications (Bordás et al., 2015; Baksa et al., 2019).

Patched neurons were labeled with biocytin and slices were fixed in 4% paraformaldehyde (in 0.1 M phosphate buffer; pH 7.4; 4°C). For permeabilization, Tris-buffered saline (TBS, in mM, Tris base, 8; Trisma HCl, 42; NaCl, 150; pH 7.4) with 0.1% Triton X-100 and 10% bovine serum (60 min) was used. Slices were incubated with streptavidin-conjugated Alexa488 (1:300; Molecular Probes Inc., Eugene, OR, USA) in TBS for 90 min.

A 3-dimensional map of somata with or without M-current was drawn with NeuroLucida software (MBF Bioscience, Williston, VT, USA) by using Paxinos atlas (Paxinos and Franklin, 2013).

Data are presented as mean ± SEM. Statistical significance was determined with a 2-sample Student’s *t*-test. The level of significance was *p* < 0.05. Pearson’s correlation coefficient (R) was applied to assess correlations between parameters by plotting them against each other and by the linear fit of this dataset.

## Results

The topographical organization of M-current possessing raphe serotonergic neurons was characterized and the impact of this current on other electrophysiological properties was described by patching 56 genetically identified serotonergic neurons (*n* = 31 from the DR and *n* = 25 from the MR; Figure 1A).

**Figure 2:**
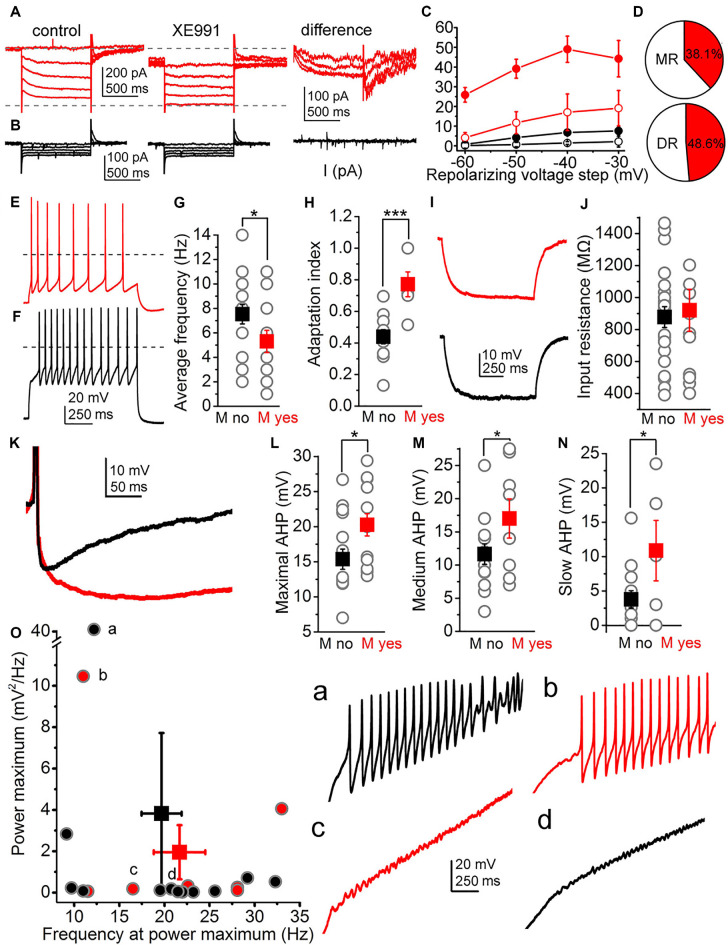
Functional characteristics of the M-current of raphe nuclei. **(A)** Representative current trace when M-current was detected (left: control current trace, middle: with the M-current inhibitor XE991, right: XE991-sensitive component). **(B)** Representative current trace when M-current is absent. The panel arrangement is similar as on panel in **(A)**. **(C)** Average relaxation currents in the function of the repolarizing voltage steps (red filled circles: M-current exists, control; red hollow circles: M-current exists, TTX; black filled circles: M-current is absent, control; black hollow circles: M-current is absent, TTX). **(D)** Proportion of neurons with M-current (red area) in the median and dorsal raphe. **(E,F)** Voltage traces elicited by 100 pA depolarizing current injections from a cell possessing (**E**, red) and from another one lacking M-current (**F**, black). **(G,H)** Statistical comparison of the average firing rate **(G)** and the adaptation index **(H)** of cells possessing (red squares) and lacking (black squares) M-current (gray circles: individual data; *p < 0.05; ***p < 0.001). **(I)** Representative voltage traces elicited by −30 pA hyperpolarizing current step on an M-current possessing (red) and –lacking neuron (black) used for calculation of input resistance. **(J)** Statistical comparison on input resistances from neurons lacking (black) and having M-current (red; gray circles: individual data). **(K)** Two superimposed representative traces of afterhyperpolarization (AHP) from neurons possessing (red) and lacking M-current (black). **(L–N)** Statistical comparison of the maximal **(L)** medium **(M)** and slow **(N)** afterhyperpolarization amplitudes (with the same arrangement as on panels **(G,H)**. **(O)** Power maxima plotted against frequency values belonging to them (black: no M-current, red: M-current is present; gray circles filled with black or red color: individual data). Letters **(a–d)** indicate individual data and representative voltage traces belonging to them (black: no M-current, red: M-current is present).

Neurons with relaxation current greater than 20 pA at −40 mV repolarizing step were considered as ones possessing M-current. In these cases (*n* = 24), holding current recorded at −20 mV was 153.94 ± 23.28 pA. Relaxation current was 25.9 ± 3.7 pA recorded at −60 mV repolarizing step; 39.16 ± 4.8 pA at −50 mV; 49.04 ± 6.64 pA at −40 mV and 44.26 ± 9.24 pA at −30 mV, respectively. When 20 μM XE991, M-current specific inhibitor was administered, the holding current was reduced to 120.78 ± 46.1 pA (*p* = 0.2), the relaxation current at −60 mV was 4.14 ± 2.64 pA (*p* = 0.0002), 11.72 ± 5.6 pA at −50 mV (*p* = 0.0033), 17.06 ± 9.27 pA at −40 mV (*p* = 0.013) and 19.14 ± 9 pA at −30 mV (*p* = 0.07; Figures 1B, 2A,C).

In neurons lacking M-current (*n* = 25), holding current was 62.4 ± 9.12 pA at −20 mV; while relaxation current was 0.75 ± 0.32 pA at −60 mv, 4.1 ± 0.1 pA at −50 mv, 6.8 ± 1.18 pA at −40 mv and 7.6 ± 1.8 pA at −30 mv, respectively. Recorded currents were not altered significantly by XE991; since the holding current was 39.05 ± 9.38 pA at −20 mv (*p* = 0.12), 0.17 ± 1.42 pA at −60 mv (*p* = 0.45), 0.66 ± 1.12 pA at −50 mV (*p* = 0.24), 1.46 ± 0.74 pA at −40 mv (*p* = 0.22) and 2.17 ± 2.77 pA at −30 mV (*p* = 0.28; Figures 2B,C) in the presence of XE991.

There was no significant difference between the M-current amplitudes of DR and MR serotonergic neurons (DR: 53.51 ± 9.31 pA, *n* = 16; MR: 38.8 ± 3.11 pA, *n* = 7; at −40 mv; *p* = 0.15). 38.1% (8 from 21) of MR serotonergic neurons had M-current, whereas this proportion was somewhat lower in the DR (48.6%; 17 from 35; Figure 2D). In the lateral wings of the dorsal raphe, even more neurons possessed M-current (60%, 6 from 10).

Next, we sought correlations between the presence of M-current and other electrophysiological parameters. We found that M-current can determine average firing rate and spike frequency adaptation. The average firing rate elicited by 100 pA depolarizing step was 5.3 ± 0.9 Hz for neurons possessing M current (*n* = 13) and 7.56 ± 0.84 Hz for those lacking M-current (*n* = 16; *p* = 0.04). Similarly, spike frequency adaptation, quantified by the adaptation index (AI), was also significantly different between the two groups. In neurons having M-current, the adaptation index was 0.77 ± 0.08 (*n* = 6), whereas this parameter was 0.44 ± 0.04 in those without M-current (*n* = 11; *p* = 0.0006; Figures 1E,F, 2E–H).

The relationship between M-current and AHP was also investigated by comparing M-current to maximal, medium, and slow AHPs. Maximal AHP amplitude was 20.27 ± 1.6 mV in the neurons possessing M-current (*n* = 13) while 15.37 ± 1.44 mV (*n* = 16) in neurons lacking M-current (*p* = 0.015). Medium AHP amplitude recorded 100 ms after the AP peak potential was 17.01 ± 2.95 mV in neurons possessing M-current, while in neurons lacking M-current this parameter was 11.65 ± 1.54 mV (*p* = 0.046). Slow AHP measured 300 ms after the AP peak was 10.87 ± 4.39 mV in neurons having M-current and 3.77 ± 1.25 mV in those without M-current (*p* = 0.024; Figures 1C, 2I–J).

Certain electrophysiological parameters seemed to be independent of the M-current. The input resistance of cells having M-current was 966.54 ± 118.14 MΩ, while the same parameter was 841.5 ± 64.74 MΩ for the ones lacking M-current, respectively (*p* = 0.16; Figure 1E).

Serotonergic raphe neurons possessed TTX-resistant high threshold membrane potential oscillations with wide frequency and amplitude ranges. The presence or absence of M-current did not seem to influence these parameters. The oscillatory frequency at the power maximum was 21.67 ± 2.86 Hz for cells having M-current and 19.69 ± 2.21 Hz for lacking M-current (*p* = 0.29), whereas the power maximum was 1.95 ± 1.31 mv^2^/Hz for cells with and 3.82 ± 3.39 mv^2^/Hz for ones without M-current (*p* = 0.33; Figures 1G,H).

**Figure 3:**
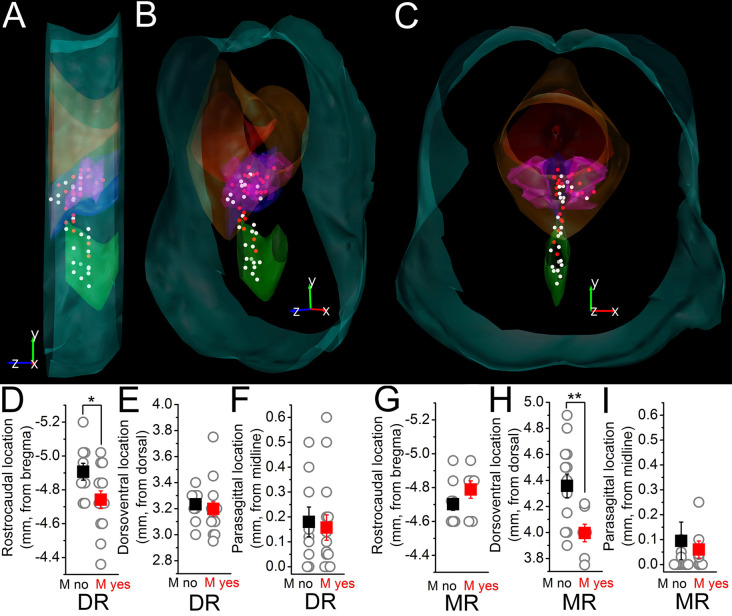
Topographical distribution of the M-current of raphe nuclei. **(A–C)** The topographical organization of the M-current possessing neurons in the raphe nuclei. **(A)** Lateral view. **(B)** Forty five degree rotation towards the front view. **(C)** Front view from caudal. White dots: somata lacking M-current. Red dots: somata with M-current. Light blue contours brainstem surface; red: aqueduct; orange: periaqueductal gray; blue: DR; purple: DR, lateral wing; green: MR. **(D–F)** Comparison of the rostrocaudal **(D)** dorsoventral **(E)** and parasagittal **(F)** locations of somata in the DR on which M-current was detected (red squares) with those where M-current was absent (black squares). **(G–I)** Comparison of the rostrocaudal **(G)** dorsoventral **(H)** and parasagittal **(I)** locations of somata in the MR with the same arrangement as in **(D–F)**. *p < 0.05; **p < 0.01.

The topographical organization of neuronal somata lacking or possessing M-current was also evaluated (Figures 3A–C). Neurons possessing M-current are located rostrally in the DR with a greater likelihood (-4.74 ± 0.05 mm from the bregma for the ones having and -4.9 ± 0.05 mm for the ones lacking M-current, *p* = 0.02) and rather located dorsally in the MR (3.99 ± 0.07 mm from the top for the ones having and 4.36 ± 0.09 mm for the ones lacking M-current; *p* = 0.007; Figures 3D–G).

**Figure 4:**
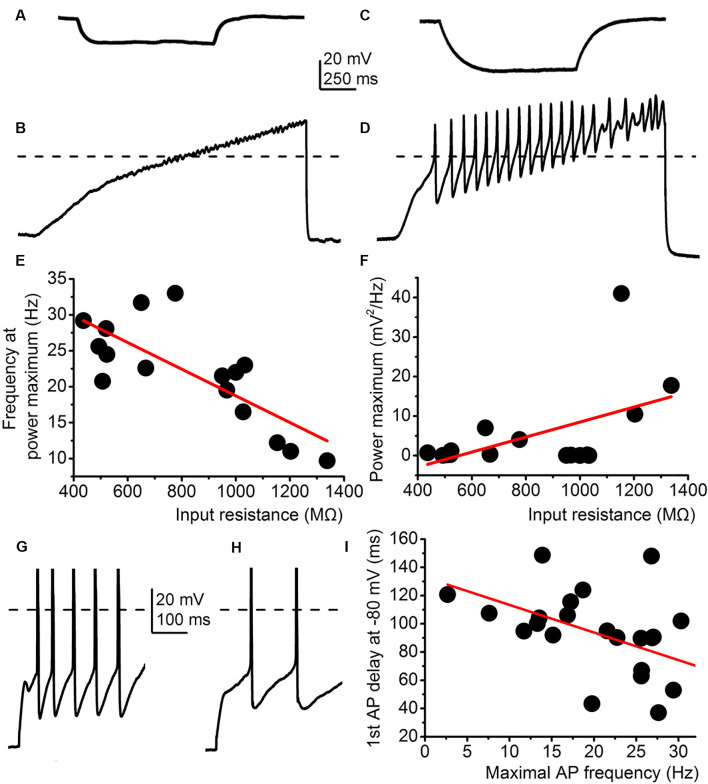
Correlations between electrophysiological parameters independent from the M-current. **(A–F)** The input resistance correlates with the parameters of high threshold membrane potential oscillations. **(A)** Representative voltage trace elicited by −30 mV hyperpolarizing current injection. **(B)** High threshold membrane potential oscillations from the same cell as on panel **(A)**. **(C)** Representative voltage trace with −30 mV hyperpolarizing current injection from another cell. **(D)** High threshold membrane potential oscillations from the same cell as on panel **(C)**. **(E–F)** Correlations between the input resistance and oscillatory frequency **(E)** and oscillatory power maximum **(F)**. **(G,H)** Two representative voltage traces where the delay is short and the maximal firing rate is high **(G)** and another one with a long delay and low maximal firing **(H)**. **(I)** Correlations of the two parameters described above with a similar arrangement seen on panels **(E,F)**.

Further correlations between electrophysiological parameters besides M-current were also found. First, the frequency of high threshold membrane potential oscillations was inversely proportional to the input resistance (*R*^2^ = 0.57), whereas power amplitude was directly proportional to the input resistance (*R*^2^ = 0.21; Figures 4A–F) in those cases when the whole trace was included in the analysis. Delay of the first action potential from the onset of the depolarizing impulse from −80 mV was inversely proportional to the maximal firing rate (*R*^2^ = 0.24; Figures 4G,I).

## Discussion

We showed that M-current positive neurons are topographically organized: in the DR, they are located rostrally; whereas M-current possessing somata are dorsal in the MR. M-current determines firing frequency, spike frequency adaptation, and the amplitude of the AHP of serotonergic neurons. High threshold membrane potential oscillations also correlate with the input resistance, and the delay of the first action potential is inversely proportional to the maximal firing rate.

M-current contributes to several electrophysiological parameters, such as neuronal excitability, AHP, and spike frequency adaptation. Although these correlations were presented in various brain structures (Koyama and Appel, 2006; Tzingounis et al., 2010; Nigro et al., 2014; Bordás et al., 2015), these have not been demonstrated on raphe serotonergic neurons. As expected, we were able to show that AHP and spike frequency adaptation is influenced by the M-current on these neurons as well.

High threshold membrane potential oscillations are under the regulation of M-current on mesopontine cholinergic neurons (Bordás et al., 2015). To the best of our knowledge, we presented for the first time that high threshold oscillations are present on raphe serotonergic neurons. In contrast with our findings on the PPN, this phenomenon is not affected by the presence or absence of M-current or any related electrophysiological parameters.

Several studies demonstrated that DR and MR serotonergic neurons are not homogenous but rather form different subgroups in terms of development, gene expression, projection, neurochemical markers, as well as *in vitro* and *in vivo* electrophysiological properties.

Developmentally, raphe serotonergic neurons arise from six rhombomeres. Subsequently, neurons with different origins have distinct gene expression patterns. Raphe serotonergic neurons form 11 transcriptomic clusters partially based on differences in expression of genes encoding ion channels (Okaty et al., 2015; Ren et al., 2019). Genetically identified subgroups of serotonergic neurons can be distinguished by different ion channel subunit expression.

MR and DR, as well as subregions of DR (including the lateral wing) project to different brain regions, and these projections have topographical organization along the dorsoventral axis (Hale and Lowry, 2011; Ren et al., 2018). Based on their projections and topography, MR and DR serotonergic neurons differ in firing pattern and action potential morphology (Hajós et al., 2007; Crawford et al., 2010; Fernandez et al., 2016). We revealed a topographical distribution of M-current possessing serotonergic neurons, which might also refer to common developmental origin or potential similarities in their projections.

M-current is inhibited by muscarinic acetylcholine receptor activation (Brown and Passmore, 2009); therefore, one can assume that serotonergic neurons with M-current undergo muscarinic cholinergic neuromodulation. Since it is also known that serotonin is capable of influencing M-current *via* the 5HT_2C_ receptor (Roepke et al., 2012), one can hypothesize that M-current possessing raphe neurons are subjects of serotonergic autoregulation. These neuromodulatory actions might be more pronounced in the rostral DR and the dorsal MR. As the rostral DR has a role in movement-related stress tolerance (Greenwood et al., 2003) and the MR is important for adaptation to chronic psychosocial stress (Graeff et al., 1996), neuromodulatory regulation *via* M-current might be involved in stress adaptation. These findings potentially provide further support to Zhao et al. (2017) that M-current might serve as a target for anxiolytic therapy.

## Data Availability Statement

The datasets generated during and/or analyzed during the current study are available from the corresponding author on reasonable request.

## Ethics Statement

The animal study was reviewed and approved by Committee of Animal Research of the University of Debrecen.

## Author Contributions

TB, AC and AK performed the experiments. AK, KP and BP analyzed the experiments and wrote the article. All authors contributed to the article and approved the submitted version.

## Conflict of Interest

The authors declare that the research was conducted in the absence of any commercial or financial relationships that could be construed as a potential conflict of interest.
